# Physicians’ Belief about Organ Donation

**Published:** 2011-11-01

**Authors:** A. Al-Marzouki, E. Tashkandi, M. U. Farooq

**Affiliations:** 1 *Ummul-Qurra University, Makkah, Saudi Arabia, *; 2 *Alnoor Specialist Hospital, Makkah, Saudi Arabia,*; 3 *Performance Measurement Manager, King Abdullah Medical City, Makkah, Saudi Arabia*

**Keywords:** Cardiopulmonary death, Brain death, Organ donation

## Abstract

Background: Several studies have suggested that knowledge, attitudes and determinants concerning organ donation are influenced by many factors including gender, educational level, occupation, sociodemographic status, income level, culture and religion.

Objective: To highlight the awareness of cardiopulmonary and brain death (CD and BD) among the physicians and their belief about the organ donation.

Methods: In a cross-sectional study, 15% of 1700 physicians working under the auspices of Ministry of Health in Makkah region, were selected randomly from two hospitals of Makkah city,* i.e*., Alnoor Specialist Hospital and King Abdalaziz Hospital. A self-administered questionnaire with dichotomous answers was distributed to them.

Results: Out of 185 respondents, 174 (94.1%) identified the right definition of BD and CD and 155 (83.3%) agreed organ donation. The difference among physicians to differentiate CD from BD was not significant (p=0.2).

Conclusion: Physicians had enough knowledge to differentiate CD from BD; most of them are highly positive regarding the concept of organ donation.

## INTRODUCTION

so far, numerous attempts have been made to develop strategies to increase organ donation. In spite of increasing number of available cadaveric grafts, the organ supply for transplantation continues to lag far behind the need, and waiting lists are still growing [1, 2]. Some studies have suggested that knowledge, attitudes and determinants concerning this issue are influenced by many factors including gender, educational level, occupation, sociodemographic status, income level, culture and religion [2-4]. Shortage of cadaveric organs for transplantation is a universal problem [4, 5]. Although people generally express favorable views towards organ donation, very few actually agree to donate before they die or agree to have family members’ organs donated upon their deaths [6, 7]. The lack of organ donation is a major limiting factor in transplantation in most countries [1-7]. Efforts to increase donation rates have included public awareness and professional educational programs, and a law that requires physicians to request families donate the organs of their deceased or dying relatives [3-5]. Yet, public health attitudes to cadaveric organ donation and transplantation are a major public health problem and of importance, since prior consent of the donor or their close relative at the time of death forms the basis for cadaveric organ donation in most countries [1-7].

Keeping in mind the above-mentioned details, we aimed our survey at the physicians who could be a very effective source of motivation for general population towards organ donation. It is a matter of proper communication. Our survey intended to explain physicians’ knowledge about differentiation of cardiopulmonary death (CD) and brain death (BD), and their attitudes towards organ donation.

## MATERIAL AND METHODS

A cross-sectional study involving physicians (consultants, specialists, residents and interns) working under the auspices of Ministry of Health in Makkah, Saudi Arabia, has been conducted. The total study population was 1700 physicians. The study sample included 15% of these physicians (n=255) selected at random and who aged between 24 and 65 years. The subjects were selected randomly from two hospitals of Makkah City, *i.e*., Alnoor Specialist Hospital, Makkah, and King Abdalaziz Hospital, Makkah. A self-administered questionnaire consisting of some personal questions followed by three leading questions were distributed to participants. The questionnaire was mainly focused on differentiation between CD and BD and agreement about organ donation. Two questions were about the definition of CD and BD, respectively, with dichotomous answers, *i.e*., “CD” and “BD.” The first question was that “if a person has irreversible cessation of circulatory and respiratory functions, *i.e*., no pulse, low blood pressure and apnea is considered as …” The second question was that “if a person has irreversible cessation of the functions of the entire brain, including the brainstem with flat EEG is considered as …” [8]. The third question was that “if you learn that a patient is BD and you have a written consent from him or his first relative, do you agree with his organ donation?” This question had also a dichotomous answer of “yes” or “no.” The survey has been conducted in December 2009. Every questionnaire began with a page describing in detail the survey and its objectives a consent from to be signed by respondents. Data were analyzed by SPSS ver 10 (SPSS Inc., Chicago, IL, USA).

The Institutional Review Boards of Alnoor Specialist Hospital and King Abdul Aziz Hospital, Makkah, granted us permission to conduct this survey.

## RESULTS

Out of 255 questionnaires distributed, 185 were completed and returned—hence, a response rate of 72.5% with male predominance (n=114, 61.6%). The number of consultants, specialists, residents, and interns participated were 37 (20%), 45 (24.3%), 58 (31.4%), and 45 (24.3%), respectively. Of 185 studied physicians, 174 (94.1%) identified the right definition of BD and CD, and 155 (83.3%) agreed with organ donation (Table 1).

**Table 1:** Knowledge of participants of differences between CD and BD, and their agreement with organ donation

Subjects’ Rank	Participants		BDI*		CDI†		ODA‡	
	n	%	n	%	n	%	n	%
Consultant	37	20.0	37	100.0	37	100.0	27	73.0
Specialist	45	24.3	43	95.6	42	93.3	38	84.4
Residents	58	31.4	54	93.1	53	91.4	50	86.2
Interns	45	24.3	40	88.9	42	93.3	40	88.9
Total	185	100.0	174	94.1	174	94.1	155	83.8

* BDI: Brain death identification

† CDI: Cardiopulmonary death identification

‡ ODA: Organ donation agreement

No difference was found in physicians’ categories regarding to the identification of correct definitions for CD and BD and their attitudes towards organ donation.

## DISCUSSION

We found that the majority of the clinicians regardless of their designation and experience could correctly differentiate BD from CD. Besides, most of them are in favor of organ donation. Most of the surveys so far have been conducted on this issue were mainly focused on general population which mostly revealed lack of information among studied people. 

A cross-sectional study/survey was conducted in Qatar to determine the knowledge, attitudes, awareness, and determinants of organ donation and transplantation. The study had a response rate of 81.5% with majority of males (67.8%). On the other hand, 70.4% of female participants were aware of organ donation and transplantation. Sixty-one percent of males and 70.4% of females were against importing organs. Male respondents generally less often accepted organ donation than females. This study concluded that intense efforts to improve public awareness and knowledge about organ donation and transplantation are necessary to maximize donation and the overall success of transplantation [9].

Another study conducted in Riyadh to evaluate the factors affecting the knowledge and attitudes of Saudi Arabian population towards organ donation and transplantation. They found that 91.1% of respondents were aware of the need for organ donation and 97.7% knew that organ donation could save lives while 23.7% were unaware of Fatwa about organ donation. Forty-two percent agreed to donate their organs after death. It was concluded that there was a need for proper information dissemination. It was suggested a multidisciplinary approach by government support backed by strong recommendations from religious authorities [10].

A study performed in Riyadh also emphasized on the difference in knowledge and attitude of urban *vs*. rural population towards organ donation. They found that urban population was more familiar with organ donation and has more willingness to donate organs than rural people. The majority of both community people emphasized on the importance of delivering the awareness of organ donation by health care providers. Finally, it was concluded that they had lack of information about this critical issue. Health care providers, local mass media, and educational institutions should provide compulsory information and encouragement to people about the importance of organ donation [11]. Daar highlighted the importance and permission of organ donation in various religions, especially Islam [12]. 

In conclusion, we found that physicians at different levels of expertise are aware of differences between CD and BD, and they agreed that organ donation in patients with BD is acceptable even among Muslims.
